# A case of Usher syndrome type IIA caused by a rare *USH2A* homozygous frameshift variant with maternal uniparental disomy (UPD) in a Chinese family

**DOI:** 10.1111/jcmm.15405

**Published:** 2020-05-25

**Authors:** Jiewen Fu, Shiyi Shen, Jingliang Cheng, Hongbin Lv, Junjiang Fu

**Affiliations:** ^1^ Key Laboratory of Epigenetics and Oncology The Research Center for Preclinical Medicine Southwest Medical University Luzhou China; ^2^ Department of Ophthalmology The Affiliated Hospital of Southwest Medical University Luzhou China

**Keywords:** frameshift mutation, homozygosity mapping, short tandem repeat, uniparental disomy (UPD), *USH2A* gene, usher syndrome type IIA, whole exome sequencing (WES)

## Abstract

Usher syndrome encompasses a group of genetically and clinically heterogeneous autosomal recessive disorders with hearing deficiencies and retinitis pigmentosa. The mechanisms underlying the Usher syndrome are highly variable. In the present study, a Chinese family with Usher syndrome was recruited. Whole exome sequencing (WES), Sanger sequencing, homozygosity mapping, short tandem repeat (STR) analysis and segregation analysis were performed. Functional domains of the pathogenic variant for *USH2A* were analysed. We identified a homozygous frameshift variant c.99_100insT (p.Arg34Serfs*41) in the *USH2A* gene in the proband that showed discordant segregation in the father. Further homozygosity mapping and STR analysis identified an unusual homozygous variant of proband that originated from maternal uniparental disomy (UPD). The p.Arg34Serfs*41 variant produced a predicted truncated protein that removes all functional domains of USH2A. The variant was not included in the 1000 Human Genomes Project database, ExAC database, HGMD or gnomAD database, but was included in the ClinVar databases as pathogenic. Although USH2A is an autosomal recessive disease, the effects of UPD should be informed in genetic counselling since the recurrence risk of an affected child is greatly reduced when the disease is due to the UPD mechanism. To test potential patients, WES, combined with STR analysis and homozygosity mapping, provides an accurate and useful strategy for genetic diagnosis. In summary, our discoveries can help further the understanding of the molecular pathogenesis of Usher syndrome type IIA to advance the prevention, diagnosis and therapy for this disorder.

## 1 INTRODUCTION

Usher syndrome consists of a group of genetically and clinically heterogeneous autosomal recessive disorders with sensorineural hearing deficiencies and progressive retinitis pigmentosa (RP). Diseases under the umbrella term Usher syndrome include Usher syndrome type I, II and III.^1^, ^2^ Usher syndrome type II includes USH2A, USH2C and USH2D. Usher syndrome type IIA (USH2A locus, OMIM 276901) is caused by mutations of the *USH2A* gene (OMIM 608400).^3^ This gene maps to the chromosome 1q41 and encodes a protein containing 5202 amino acids that contain a pentaxin motif, laminin EGF motifs and numerous fibronectin type III domains.^4^ The protein is localized in the basement membrane and has a vital role for development and homeostasis in the inner ear and retina.

Homozygosity has long been known to be related to rare often devastating Mendelian disorders and imprinting diseases.^5^ Uniparental disomy (UPD) is the inheritance of both copies of one chromosome from only one parent, without the inheritance of a representative copy from the other parent.^6^, ^7^ Euploidy can result from aneuploid gametes if monosomic rescue, trisomic rescue or gametic complementational restore of normal ploidy occur during early human development. Detecting UPD is a practical diagnostic approach for rare Mendelian disorders and imprinting disorders caused by homozygosity.^7^, ^8^, ^9^

The relationship between the variants in the Usher syndrome‐associated genes and the resultant Usher syndrome phenotypes in the patients is highly variable. The causality and genetic mechanism of Usher syndrome type IIA have not been well documented. In the present study, we identified a rare homozygous frameshift mutation in the gene *USH2A* that originated from maternal UPD by whole exome sequencing (WES) and homozygosity mapping in a Chinese pedigree with Usher syndrome.

## 2 MATERIALS AND METHODS

### 2.1 Pedigree construction, sample collection and DNA isolation

**Figure 1:**
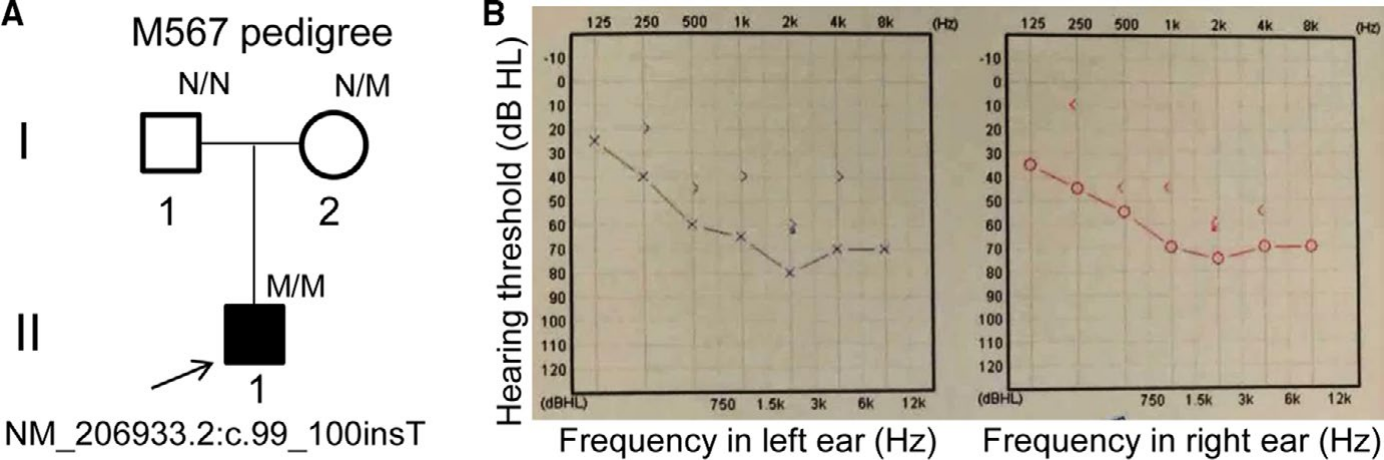
A M567 pedigree with Usher syndrome type IIA and the pure‐tone audiograms. A, M567 pedigree with Usher syndrome type IIA. Normal individuals are shown as clear circle (female) or square (male). The filled square indicates the proband (II: 1, arrow) with the homozygous mutation of the *USH2A* gene: NM_206933.2:c.99_100insT. “N” indicates the wild‐type allele, whereas “M” indicates the mutant allele. B, The pure‐tone audiograms of the proband (II:1). The dark blue “X” line shows the left ear's results from an air conduction test, whereas the red “O” line shows the right ear's results. The dark blue “>” line shows the left ear's results from a bone conduction test, whereas the red “<” line shows the right ear's results. An air‐bone gap is great than 10 dB

The M567 pedigree consisted of a proband and his parents (Figure 1A; I:1, M565; I:2, M566; II:1, M567). Pure‐tone audiometry testing of the proband was performed to determine hearing thresholds at frequencies 0.125, 0.25, 0.5, 1, 2, 4 and 8 kHz.^1^ Depending on the severity, hearing loss can be categorized as mild (26 ~ 40 dB), moderate (41 ~ 55 dB), moderate‐severe (56 ~ 70 dB), severe (71 ~ 90 dB) or profound (<90 dB).^10^ Written informed consent from the participants or guardians following the Declaration of Helsinki was obtained. Blood samples were taken, and genomic DNA was isolated from this family.^11^, ^12^ DNA from blood samples was taken from healthy controls (n = 100).^13^

### 2.2 Whole exome sequencing (WES) and bioinformatics analysis

WES analysis was performed on the proband M567 gDNA (I:1) (Genmed, Inc).^13^, ^14^, ^15^, ^16^, ^17^ Library preparation including fragmented DNA, DNA end filling, joint addition, PCR enrichment and library quantification was performed. Then, enriched target fragments or regions were obtained by hybridization, washing, DNA elution, PCR amplification and purification. Sequencing was performed on the Illumina X‐ten (Illumina, Inc) after cluster generation, and raw FASTQ data were collected. Then, the FASTQ data were compared to a reference genome (GRCh38/hg38) to obtain the Bam file. After identifying the variations of single nucleotide polymorphism (SNP), Indel (insertion/deletion) and CNV (copy number variant) in the Bam file, the VCF (variant call format) file was obtained. The mutation sites in VCF file were annotated, and this annotated file was obtained. Variant screening was performed by combining with clinical symptoms and genetic patterns.^15^, ^18^, ^19^ Conserved domains of CDD (NCBI's conserved domain database) in USH2A protein (NP_996816.2) were searched through the online program (https://www.ncbi.nlm.nih.gov/Structure/cdd/wrpsb.cgi).^20^, ^21^, ^22^

### 2.3 Sanger validation and segregation analysis

PCR amplification was performed for mutation validation.^13^, ^23^ Primer pairs M567‐USH‐99 were designed using the Primer3 program with genomic DNA sequences containing the NM_206933.2 variant: c.99_100insT in the *USH2A* gene (Table 1). PCR amplification was performed using the aforementioned primer pair USH2A‐99, and the amplified PCR products were then sequenced using the Sanger method on an ABI‐3500DX sequencer ^24^, ^25^ using primer USH2A‐99L (Table 1). Ethnically matched unrelated control samples were also sequenced as described above. Testing of the variant in the mother and father ensued.

**Table 1:** The sequences of *USH2A* PCR primers for PCR amplification

Primer name	Left primer	Sequence (5′‐3′)	Right primer	Sequence (5′‐3′)	Size	TM
USH2A‐99	USH2A‐99L	gaacgtgtctgcagtttcca	USH2A‐99R	tcagctttggagaaggagga	574	60

### 2.4 PCR amplification and genotyping for short tandem repeat (STR)

Short tandem repeat genotype analysis was carried out in accordance with the relevant provisions of the Technical Specification for Paternity Appraisal by China (SF/Z JD0105001‐2018) with the GoldenEye™ kit. PCR reactions were carried out according to the manufacturer's instructions using the extracted genomic DNA samples. The PCR amplification cycle was performed in an “Applied Biosystems Veriti^®^ 96‐Well Thermal Cycler” machine. Then, the amplified product was mixed with the sample mixture for capillary electrophoresis using the 3500DX Gene Analyzer (Applied Biosystems Inc).^24^ Genotype analysis for STR profiles was performed by using the software of GeneMapper^®^ ID‐X 1.5.

### 2.5 Homozygosity mapping

The homozygosity mapping analysis was performed using the Illumina ASA (Asian Screening Array) (Genmed, Inc). To do this, we used a PLINK software, Illumina chip matching reagents and an Illumina instrument (Multi‐Sample BeadChip Alignment Fixture). By applying the PLINK software, we screened loci with a missing rate < 1%, minor allele frequency (MAF) > 5%, and Hardy‐Weinberg equilibrium *P* > 1e‐3, and set one window for every 50 SNPs and slid 5 SNPs for each section with *r* ^2^ < .5 in two SNPs. If a region spanning 2.5 Mb contained continuous homozygous SNPs, then the region was considered to be a possible homozygous region (run of homozygosity, ROH). Thus, the SNPs selected using these methods were relatively independent and more common in the whole genome.^26^

## 3 RESULTS

### 3.1 Proband and clinical characteristics

The patient (Figure 1A, II: 1) was a 5‐year‐old Chinese boy. He had been clinically diagnosed with hearing loss since age 5 years. A pure‐tone audiometry testing was performed which indicated binaural moderate to severe deafness with sloping audiograms that included increased thresholds across all frequencies (Figure 1B) (left ear: 68.3 dB; right ear: 66.7 dB). An air‐bone gap value was great than 10 dB in the proband (Figure 1B). Thus, this proband should show a mixed deafness (both sensorineural and conductive defects). The proband reported normal vision and declined ophthalmic examination. Both parents had normal hearing and vision. Thus, the proband may potentially has Usher syndrome type with an autosomal recessive pattern.

### 3.2 A homozygous variant c.99_100insT (p.Arg34Serfs*41) of the proband causes Usher syndrome type IIA

**Figure 2:**
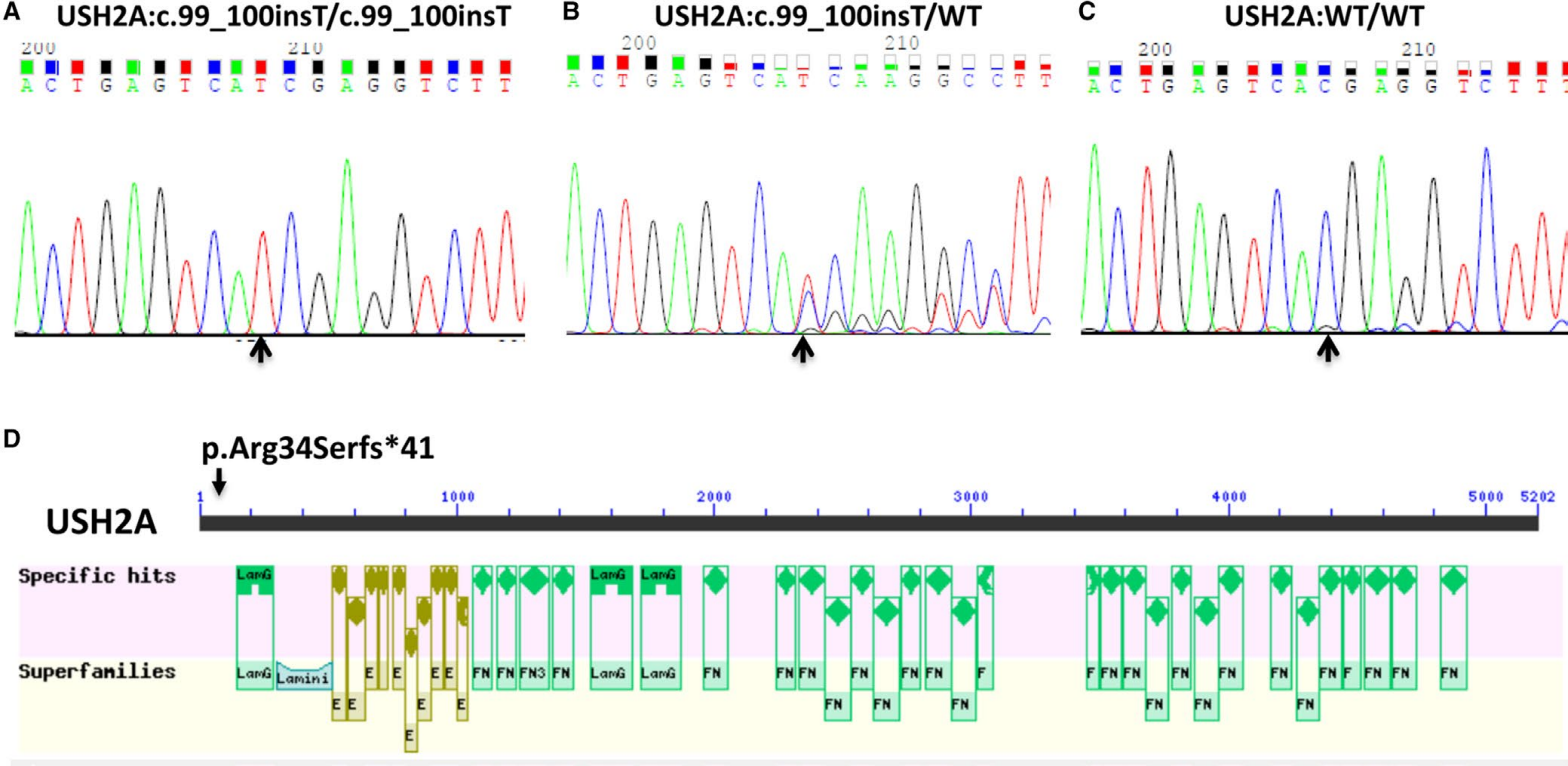
Electropherogram profiles for Sanger sequencing, USH2A structure and mutant position. A‐C, indicate the sequenced results in II: 1 (homozygous mutant type), I: 2 (heterozygous mutant type), I: 1 (wild type) of variant c.99_100insT in *USA2A*, respectively. The arrows show the mutant position. “WT” indicates wild type. D, USH2A domains and the mutant position. The variant p.Arg34Serfs*41 of USH2A is indicated in the (D), where the arrow indicates the mutant position. Note: “LamG” indicates Laminin EGF domain, “Laminin” indicates Laminin N‐terminal (Domain VI), “E” indicates Laminin‐type epidermal growth factor‐like domain, and “FN” or “F” indicates Fibronectin type 3 domain

WES identified a homozygous frameshift mutation c.99_100insT with a single nucleotide homozygous insertion in exon 1 in the *USH2A* gene (NM_206933.2), leading to an amino acid exchange from arginine (Arg) to serine (Ser) at the position 34, and a frameshift with another 41 amino acids following a stop codon (p.Arg34Serfs*41) in the USH2A protein (NP_996816.2) (Figure 1A, II 1) (Table. 2). The variant c.99_100insT was verified by Sanger sequencing (Figure 2A). This variant was absent in the 100 ethnically matched normal hearing and vision controls. The USH2A protein in *H sapiens* contains a LamG‐like jellyroll fold domain, laminin‐type EGF‐like domains, laminin G domains, laminin N‐terminal (Domain VI) and many fibronectin type 3 domains (Figure 2D). The variant c.99_100insT (p.Arg34Serfs*41) causes a loss of all functional domains (Figure 2D). Thus, our studies indicate that the *USH2A* pathogenic, homozygous variant c.99_100insT (p.Arg34Serfs*41) should cause Usher syndrome type IIA in the proband in this Chinese family. The variant was not included in the 1000 Human Genome Project, ExAC, HGMD and gnomAD databases, but it was included in the database of ClinVar (accession number: VCV000520636.1, website: https://www.ncbi.nlm.nih.gov/clinvar/variation/520636/) along with its pathogenicity^27^ (Table. 2).

**Table 2:** Characteristics of *USH2A* variant and analysis of disease‐causing effects of proband

Gene	Exon	Variation				ExAC
		Nucleotide	Protein	Type	Status	
USH2A	1	c.99_100insT	p.Arg34Serfs*41	frameshift	maternal homozygous	Novel

*Stop codon.

Abbreviations: c, variation at cDNA level; ExAC, Exome Aggregation Consortium; p, variation at protein level; USH2A, Usherin.

### 3.3 Discordant segregation of the c.99_100insT variant in the proband's father

Sanger sequencing was performed for the co‐segregation analysis. As expected, the heterozygous mutation was identified in the proband's mother (Figure 2B), M566, I:2). But surprisingly, the proband's father (M565, I:1) had a wild‐type genotype (Figure 2C) instead of the expected heterozygous genotype. Thus, this variant c.99_100insT (p.Arg34Serfs*41) was not inherited from the father.

### 3.4 Homozygous variant c.99_100insT is due to upd from proband's mother

**Figure 3:**
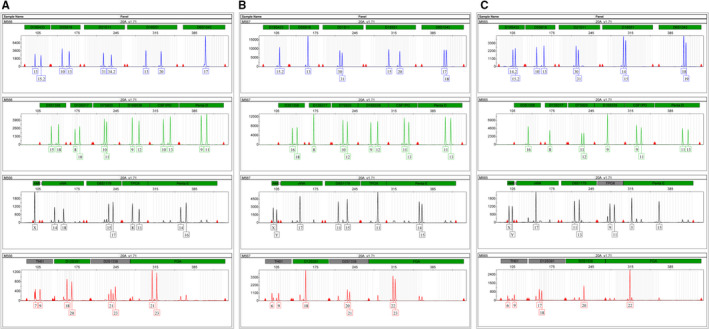
Authentication of suspected father by STR (short tandem repeat) genotypes from the family of M567 pedigree. A, An electropherogram of STR genotypes from proband's mother I:2. B, An electropherogram of STR genotypes from the proband II:1. C. An electropherogram of STR genotypes from proband's father I:1. The “Y” axis indicates the values of RFU (relative fluorescence units), whereas “X” axis indicates the STR markers for loci

Given that the mutant allele should come from the proband's father or arise de novo, we perform STR analysis to confirm the paternity by using 20 STR markers, including 19 autosomal markers and an Amelogenin gender marker. The results showed that, with the exception of the locus vWA mutation from the mother (I:2), all STR alleles in the proband II:1 were inherited from I:1 and I:2 with a combined paternity index (CPI) of 5.7541 × 10^8^ (>1 × 10^5^) (Figure 3, Table. 3). Thus, M565 (I:1) was confirmed as the biological father of proband (M567, II:1). We should point that the allele of “17” of vWA marker in the son is mostly likely inherited from his mothers’ allele of “18”.

**Table 3:** STR analysis results for M567 pedigree

STR locus	M566(m)		M567(s)		M565(f)		Calculation	PI value
AMEL	X		X	Y	X	Y		
D19S433	13	15.2	15.2	15.2	14.2	15.2	1/2p	3.02
D5S818	10	13	13	13	10	13	1/2p	3.52
D21S11	31	34.2	30	31	30	31	1/2p	1.79
D18S51	13	20	15	20	14	15	1/2p	2.92
D6S1043	17	17	17	18	18	19	1/2p	2.63
D3S1358	15	18	16	18	16	16	1/p	3.05
D13S317	8	10	8	8	8	8	1/p	3.48
D7S820	10	11	10	12	11	12	1/2p	1000.00
D16S539	9	12	9	12	9	9	1/(p + q)	2.04
CSF1PO	10	13	11	13	9	11	1/2p	2.01
Penta D	9	11	11	13	11	13	1/2p	4.94
vWA	14	18	17	17	17	17	μ/2P	0.00
D8S1179	15	17	11	15	11	13	1/2p	5.34
TPOX	8	11	11	11	9	11	1/2p	1.67
Penta E	14	16	14	15	5	15	1/2p	6.65
TH01	7	9	6	9	6	9	1/2p	5.04
D12S391	18	20	18	18	17	18	1/2p	2.63
D2S1338	21	23	20	21	18	20	1/2p	4.10
FGA	21	23	22	23	22	22	1/p	5.36
							CPI	5.7541E + 08

Note

μ = 0.0005; p and q are the frequencies of the alleles.

Abbreviations: CPI, Combined Paternity Index; f, father; m, mother; PI, Paternity Index; s, proband; STR, short tandem repeat.

**Figure 4:**
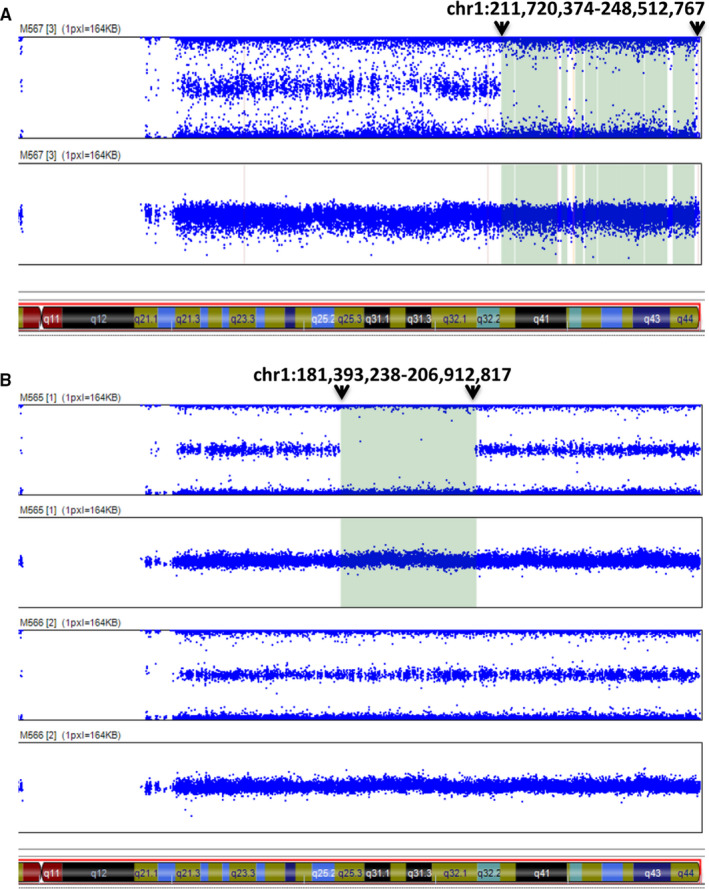
Allele frequency distribution in the long arm of chromosome 1. A, M567 (II:1, proband). B, M565 (father I:1, upper panel) and M566 (mother I:2, lower panel). The green box area is ROH (run of homozygosity). Arrows indicate the boundaries of ROHs

To further investigate the resultant mechanism for the homozygous variant c.99_100insT in the proband, homozygosity mapping was performed by SNP array. The results showed ROHs spanning tens of Mb long on chromosome 1 in the proband and father but of different regions. As shown in Figure 4, the starting and ending position of the ROH region of the son (M567) is chr1:211 720 374‐248 512 767 (Figure 4A), whereas the starting and ending position of the ROH region of the father (M565) is chr1:181 393 238‐206 912 817 (Figure 4B, top panel), and no ROH was found in the mother (M566) (Figure 4B, bottom panel). Further examination of the SNP genotype determined that the ROH of the proband (M567 sample) is actually a 36.79 Mb UPD (chr1:211 720 374‐248 512 767) of maternal origin that includes the entire *USH2A* gene. Thus from these data, we demonstrated that the homozygous variant c.99_100insT in the proband is due to maternal UPD, not de novo origin or a heterozygous micro‐deletion of the proband's father.

## 4 DISCUSSION

In this study, we described a homozygous, pathogenic frameshift variant NM_206933.2:c.99_100insT (p.Arg34Serfs*41) in the *USH2A* gene in a Chinese pedigree. This variant removed all functional domains and elucidated the genetic roles of the *USH2A* mutant allele in this family inflicted with Usher syndrome type IIA. The variant was not included in the 1000 Human Genome Project, ExAC, HGMD and gnomAD databases, but was included in the ClinVar database along with its pathogenicity in this ClinVar database (https://www.ncbi.nlm.nih.gov/clinvar/variation/520636/). With an accurate genetic diagnosis for Usher syndrome, it may be possible to repair or replace defective gene copies in many afflicted patients as new therapies are developed.^28^, ^29^, ^30^ Fuster‐García et al^31^ explored methods for gene editing for targeting the pathogenic mutation in fibroblasts of an USH patient bearing c.2299delG homozygous variation. Similarly, Sanjurjo‐Soriano et al^32^ used a Cas9 protein with enhanced specificity in Streptococcus pyogenes (eSpCas9) to correct two *USH2A* mutations, c.2276G > T and c.2299delG, in induced pluripotent stem cells (iPSCs) of USH/arRP patients. Furthermore, as the drug authorized Duchenne muscular dystrophy (DMD) and cystic fibrosis (CF) treatment in the USA and conditionally authorized for DMD treatment in Europe, ataluren has recently been reported to treat fibroblasts from *USH2A* mutated patient.^33^

There are five possibilities for the presence of discordant segregation in this pedigree: no paternity or sampling errors, de novo mutation, heterozygous micro‐deletion and UPD. UPD is described in roughly 3300 cases so far and has been linked to clinical phenotypes due to imprinting disorders or recessive diseases, including schizophrenia, cardiovascular disease and cognitive impairment.^5^, ^7^, ^34^ Detecting UPD is a useful diagnostic approach for uncovering rare Mendelian diseases caused by homozygosity. Genetic counselling for families with recessively inherited eye/hearing diseases should accept the possibility that an unaffected heterozygous carrier can have affected offspring homozygous for the same pathogenic variation, even if the carrier's spouse has wild‐type alleles at the same locus.^35^

The homozygous region contains the homozygous allele in the genome, which can arise through the inheritance of both alleles from either maternal or paternal origin. Typically, in this case, these two mutant alleles are given by the same ancestors. Thus, homozygosity mapping analysis can be used to determine genetic diseases caused by inbreeding and to determine single diploidy. Our STR analysis and homozygosity mapping revealed the proband's homozygous variant c.99_100insT (p.Arg34Serfs*41) is the maternal UPD with a 36.79 Mb homozygosity region on chromosome 1 containing the whole *USH2A* gene. The genotypes of the other chromosomes’ SNP markers are consistent with the patient inheriting alleles from both parents. Although Usher syndrome type IIA is an autosomal recessive disease, genetic counselling should inform UPD effects to the patients to prevent misunderstanding, since the risk of an affected child is markedly low if the disease is caused due to UPD.^36^

A pure pure‐tone audiometry testing in the proband indicated the binaural moderate to severe deafness with sloping audiograms that included increased thresholds across all frequencies (Figure 1B). An air‐bone gap value was great than 10 dB in the both ears, meaning a problem in the outer or middle ears of the proband (Figure 1B). Thus, this proband should show the mixed deafness with both sensorineural and conductive defects, not only the sensorineural deafness. It is unusual as an Usher syndrome but do show an air‐bone gap. The patient was cooperated well when did the pure‐tone audiometry testing. The proband was showed normal vision likely because of too young to develop vision impairments.

In conclusion, we have successfully identified a rare homozygous frameshift variant c.99_100insT (p.Arg34Serfs*41) with maternal UPD in the *USH2A* gene, which would cause Usher syndrome type IIA in our Chinese family. NGS,^37^ combined with STR analysis^24^ and homozygosity mapping,^38^ provides an accurate genetic diagnostic approach. Our discoveries can help elucidate the molecular pathogenesis of Usher syndrome type IIA and contribute to the genetic counselling, prevention, diagnosis, and therapy of this disorder.

## CONFLICT OF INTEREST

None.

## AUTHOR CONTRIBUTION

**Jiewen Fu:** Investigation (lead); Methodology (lead). **Shiyi Shen:** Investigation (lead). **Jingliang Cheng:** Investigation (equal); Methodology (equal); Software (equal). **Hongbin Lv:** Project administration (equal). **Junjiang Fu:** Conceptualization (equal); Funding acquisition (equal); Investigation (equal); Project administration (lead); Resources (equal); Supervision (equal); Writing‐original draft (equal); Writing‐review & editing (equal). 

## ETHICAL APPROVAL

The study has been approved by the Ethics Committee of Southwest Medical University. The informed consent form was obtained from the members of the family or guardian.

## Data Availability

All data used for the analyses in this report are available from the corresponding author on reasonable request.
